# Design and Development of Gold-Loaded and Boron-Attached Multicore Manganese Ferrite Nanoparticles as a Potential Agent in Biomedical Applications

**DOI:** 10.1021/acsomega.2c02074

**Published:** 2022-06-03

**Authors:** Okan Icten, Beril Erdem Tuncdemir, Hatice Mergen

**Affiliations:** † Department of Chemistry, Faculty of Science, Hacettepe University, Ankara 06800, Turkey; ‡ Department of Biology, Faculty of Science, Hacettepe University, Ankara 06800, Turkey

## Abstract

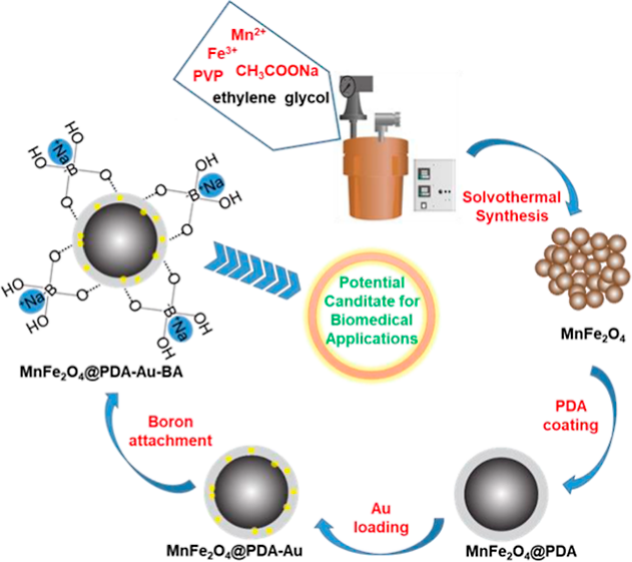

Early diagnosis and effective treatment of cancer are significant issues that should be focused on since it is one of the most deadly diseases. Multifunctional nanomaterials can offer new cancer diagnoses and treatment possibilities. These nanomaterials with diverse functions, including targeting, imaging, and therapy, are being studied extensively in a way that minimize overcoming the limitations associated with traditional cancer diagnosis and treatment. Therefore, the goal of this study is to prepare multifunctional nanocomposites possessing the potential to be used simultaneously in imaging such as magnetic resonance imaging (MRI) and dual cancer therapy such as photothermal therapy (PTT) and boron neutron capture therapy (BNCT). In this context, multi-core MnFe_2_O_4_ nanoparticles, which can be used as a potential MRI contrast agent and target the desired region in the body via a magnetic field, were successfully synthesized via the solvothermal method. Then, multi-core nanoparticles were coated with polydopamine (PDA) to reduce gold nanoparticles, bind boron on the surface, and ensure the biocompatibility of all materials. Finally, gold nanoparticles were reduced on the surface of PDA-coated MnFe_2_O_4_, and boric acid was attached to the hybrid materials for also possessing the ability to be used as a potential agent in PTT and BNCT applications in addition to being an MRI agent. According to the cell viability assay, treatment of the glioblastoma cell line (T98G) with MnFe_2_O_4_@PDA-Au-BA for 24 and 48 h did not cause any significant cell death, indicating good biocompatibility. All analysis results showed that the developed MnFe_2_O_4_@PDA-Au-BA multifunctional material could be a helpful candidate for biomedical applications such as MRI, PTT, and BNCT.

## 1 Introduction

Cancer is a disease that is the second foremost cause of death globally after cardiovascular diseases. According to the World Health Organization Cancer Report, more than 18 million people were diagnosed with cancer in 2018, and approximately 9.6 million people died due to cancer. Poor clinical outcomes are due to the lack of appropriate tools for early detection and effective cancer treatment.^1^ However, clinicians believe that early cancer detection can significantly improve the chances of successful treatment and survival.^2^ In this context, nanomaterials can offer new cancer diagnosis and treatment possibilities. In particular, multifunctional nanomaterials with diverse functions, including targeting, imaging, and therapy, are being studied extensively in a way that minimize overcoming the limitations associated with traditional cancer diagnosis and treatment.^3^ For cancer treatment, new multifunctional nanoparticles used in many new treatment methods such as photothermal therapy (PTT), magnetic hyperthermia (MH), photodynamic therapy (PDT), and boron or gadolinium neutron capture therapy (BNCT and GdNCT) have been developed to be applied in recent years apart from traditional treatments such as surgery, chemotherapy, and radiotherapy.^4−7^ In addition, the preparation of functional nanomaterials that allow simultaneous imaging, such as magnetic resonance imaging (MRI) for the correct detection of the tumor region in cancer treatment, will positively contribute to the treatment’s effectiveness.^8^ In this respect, the main aim of this study is to prepare multifunctional nanocomposites possessing the potential to be used simultaneously in imaging such as MRI and dual cancer therapy such as PTT and BNCT.

Nanoscale materials, especially functional materials containing magnetic nanoparticles, are widely used for cancer diagnosis and treatment because of unique properties such as easy synthesis and surface modification, biocompatibility, and easy control by using an external magnet.^9−11^ One of the applications of magnetic nanoparticles is their use as a *T*_2_ contrast agent in MRI, which is one of the most potent non-invasive diagnostic tools because they can effectively increase the relaxation efficiency of the water proton and accordingly shorten the relaxation time of the protons. Compared to iron oxide nanoparticles, manganese ferrite nanoparticles (MnFe_2_O_4_) are commonly used as *T*_2_ contrast agents and possess high saturation magnetization and excellent *T*_2_-negative contrast because they can be shown to be more efficient at promoting transverse relaxivity (*r*_2_).^12−15^ Although the *r*_2_ values can be increased by increasing the size of the individual particles, this causes some difficulties, such as large and mononuclear magnetic nanoparticles tend to be polydisperse and agglomerated in suspension. One option to overcome this restraint would be to place multiple magnetic cores on a larger substrate such as silica or an organic layer, resulting in an increase in the total magnetic content per particle and an improvement in high colloidal stability.^16,17^ In addition to being an MRI agent, MnFe_2_O_4_ nanoparticles have been recognized as a magnetic hyperthermia tool in cancer therapy due to the generation of heat under a magnetic field and drug carrier.^18^

PTT is a highly specific and minimally invasive cancer treatment method depending on the killing of cancer cells by achieving sufficient hyperthermia under laser irradiation compared to the traditional cancer treatment methods such as chemotherapy and radiotherapy.^19−21^ In recent years, various nanomaterials such as different kinds of gold nanostructures,^22^ carbon base nanostructures,^23^ and copper sulfur nanoparticles^24^ have been developed and studied as PTT agents as they efficiently convert optical energy into thermal energy and increase the effectiveness of photothermal ablation therapy.^25^ Among these nanostructures, gold-based nanostructures are getting more attention as a photothermal agent in PTT applications because of their low toxicity, biocompatibility, high surface plasmon resonance, and capability of further functionalization.^20,26^ One of the main challenges in PTT applications depending on the gold nanostructures is the need to accumulate enough gold nanoparticles in the tumor area to increase the thermal effect while reducing the laser intensity to avoid damage to healthy tissues.^27^ The other challenge is the cytotoxicity issue from the cytotoxic cetyltrimethylammonium bromide (CTAB) surfactant used to synthesize gold nanoparticles.^26,28,29^ Considering these challenges related to gold nanoparticles in cancer treatment, one of the main aims of the study is to combine gold nanoparticles with a magnetic core to accumulate them at the desired level in the tumor area and to use a biocompatible polymer such as polydopamine (PDA) for the synthesis of gold nanoparticles. PDA has been applied in biomedical fields due to its high biocompatibility and biodegradability^30^ in addition to catalytic applications as a carbon source.^31−33^ Additionally, thanks to the multi-core manganese ferrite nanoparticles in the synthesized hybrid material structure, it can also increase the effectiveness of the treatment as it allows imaging with MRI during PTT applications.

Previous studies have indicated that a single treatment alone cannot be sufficient to destroy especially malignant tumors owing to the individual differences between cancer patients and drug resistance of cancer cells. Therefore, more than one treatment can be simultaneously applied to the patients, creating more effects and increasing the effectiveness of the treatment.^34−36^ BNCT is an effective cancer treatment method based on ^10^B isotopes and low-energy neutron beams. The basic principle of BNCT is dependent on the accumulation of ^10^B isotopes in the cancerous region and then the excitation of these isotopes with low-energy neutrons and the destruction of the cancer cells using high-energy particles [(^4^He^2+^) and ^7^Li^3+^ ions] produced by excitation without affecting normal cells.^37,38^ Although there are some nanocomposites, which include boron and gold atoms, that have the potential to be used for the combination of BNCT and PTT methods,^20,39−41^ very few of them have been seen to be combined with the magnetic core to provide an adequate amount of boron or gold in cancer cells.^42,43^

**Figure 1:**
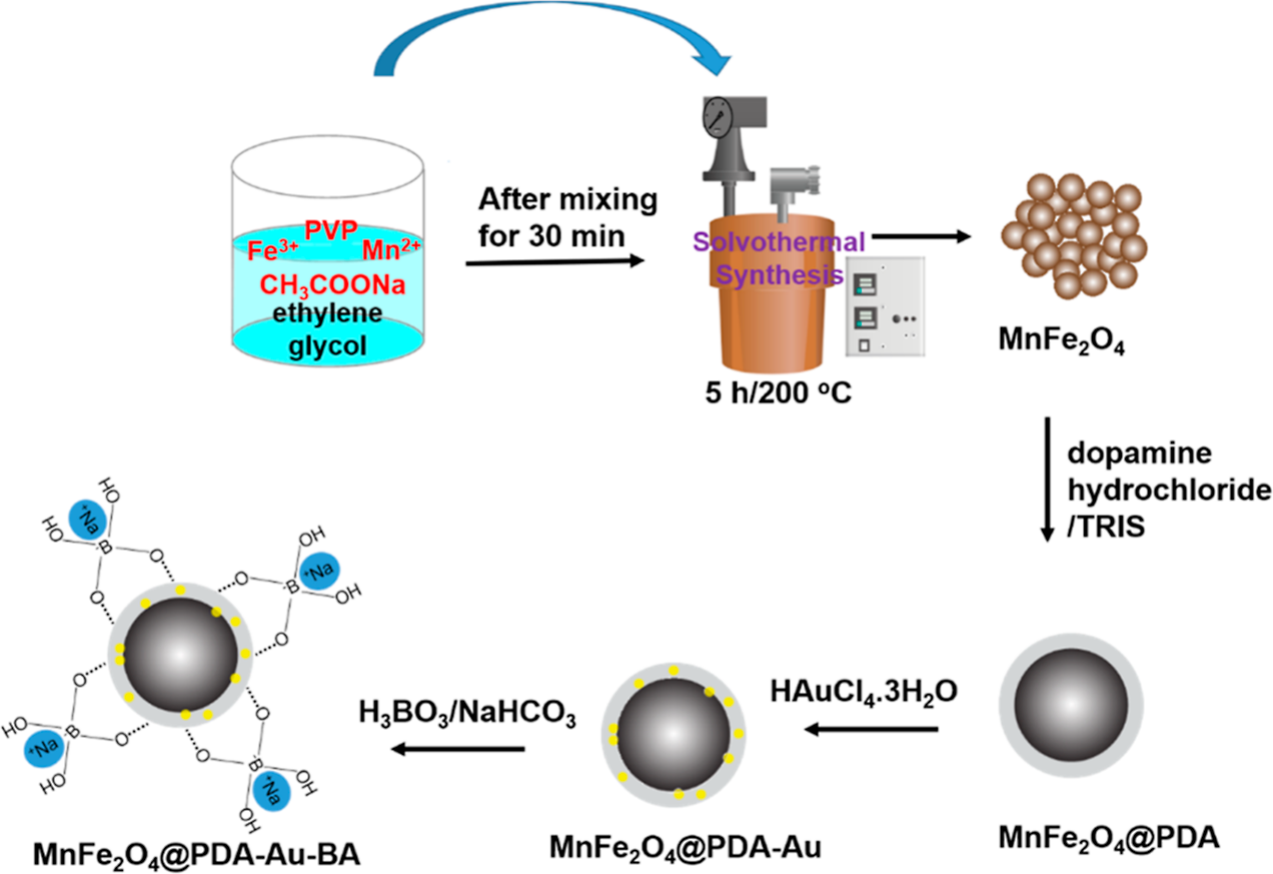
Schematic description of the synthesis of gold-loaded and boron-attached multicore manganese ferrite nanoparticles (MnFe_2_O_4_@PDA-Au-BA).

In this context, the study aims to first synthesize multi-core manganese ferrite nanoparticles, which can be used as a potential MRI contrast agent and target the desired region in the body, and then to coat these multicore nanoparticles with PDA for reduction of gold nanoparticles, binding of boron atoms on the surface, and ensuring the biocompatibility of all materials. Finally, gold nanoparticles were reduced on the surface of PDA-coated MnFe_2_O_4_, and boric acid was attached to the hybrid materials for also possessing the ability to be used as a potential agent in PTT and BNCT applications in addition to being an MRI agent (as shown in Figure 1).

## 2 Experimental Section

### 2.1 Materials

Fe(NO_3_)_3_·9H_2_O (Merck), MnSO_4_·H_2_O (Sigma-Aldrich), CH_3_COONa·3H_2_O (Merck), polyvinylpyrrolidone (PVP, average mol wt 40.000, Sigma-Aldrich), tris(hydroxymethyl)aminomethane (TRIS, Sigma-Aldrich), dopamine hydrochloride (Sigma-Aldrich), HAuCI_4_·3H_2_O (Carlo Erba), absolute ethanol (ISOLAB), NaHCO_3_ (Merck), and H_3_BO_3_ (Sigma-Aldrich) were used without any further purification.

### 2.2 Synthesis of Multicore Manganese Ferrite Nanoparticles (MnFe_2_O_4_)

MnFe_2_O_4_ nanoparticles were successfully synthesized using the solvothermal method. Fe(NO_3_)_3_·9H_2_O (3.13 g) and MnSO_4_·H_2_O (0.79 g) were dissolved in ethylene glycol (60 mL) to form a clear solution, followed by the addition of CH_3_COONa·3H_2_O (5.97 g) and PVP (0.9 g). The mixture was stirred vigorously for 30 min, filled in the reactor vessel (Parr 5500), and heated at 200 °C for 5 h. The obtained solid product was washed several times with deionized water and ethanol and dried under a vacuum oven at 70 °C for 24 h.^44^

### 2.3 Synthesis of Polydopamine-Coated MnFe_2_O_4_ Nanoparticles (MnFe_2_O_4_@PDA)

For PDA coating, 75 mg of MnFe_2_O_4_ nanoparticles was dispersed in the solution mixture of ethanol (20 mL) and aqueous solution (10 mL) of TRIS (25 mM). 15 mL of aqueous solution with dissolved dopamine hydrochloride (150 mg) was added, and the resulting mixture was stirred for 24 h at room temperature. The product was collected with an external magnet, washed with deionized water, and dried in a vacuum oven at 40 °C overnight.^45^

### 2.4 Synthesis of Gold-Loaded MnFe_2_O_4_@PDA Nanoparticles (MnFe_2_O_4_@PDA-Au)

75 mg of MnFe_2_O_4_@PDA nanoparticles was dispersed in 40 mL of gold solution (15 mg HAuCI_4_·3H_2_O) and sonicated for 5 min. The mixture was stirred for 24 h and separated using an external magnet. After being washed with deionized water several times, the final product was dried in a vacuum oven at 40 °C.^46^

### 2.5 Synthesis of Boron-Attached MnFe_2_O_4_@PDA-Au Nanoparticles (MnFe_2_O_4_@PDA-Au-BA)

27.5 mg of MnFe_2_O_4_@PDA-Au nanoparticles was dispersed in 10 mL of deionized water and sonicated for 5 min 3.78 mg of NaHCO_3_ and 2.81 mg of H_3_BO_3_ were added to the mixture, and the final mixture was stirred under a nitrogen atmosphere for 1 h. Then, the water was evaporated using a rotary evaporator, and cold acetone was added to the concentrated mixture. After waiting one night, the final product was separated and dried in a vacuum oven at 40 °C.^47^

### 2.6 Characterization

Powder X-ray diffraction (XRD) patterns were recorded with a Rigaku Ultima IV X-ray diffractometer between 2θ: 2 and 70° with 2°/min. Fourier transform infrared (FT-IR) spectra were obtained using a PerkinElmer Spectrum-One instrument. Thermogravimetric analysis (TGA) curves were obtained by using a Shimadzu DTG-60H instrument under nitrogen flow (heating rate: 10 °C/min). Scanning electron microscopy (SEM) and transmission electron microscopy (TEM) analyses were performed with a Tescan GAIA 3 instrument and a Jeol 2100F model instrument (120 kV), respectively. Room-temperature magnetization curves were recorded using the Cryogenic Limited Physical Property Measurement System (PPMS) between ±10 kOe at room temperature. B and Au amounts were determined with the ICP-OES instrument (PerkinElmer Optima 4300 DV model). Zeta potentials were measured using Malvern Nano ZS90. Absorbance at 570 nm was measured with an EnSight Multimode Plate Reader (PerkinElmer).

### 2.7 In Vitro Cell Culture Experiments

T98G is a glioblastoma cell line, and it is commonly used for cell viability assays.^48−53^ To determine the effect of MnFe_2_O_4_@PDA-Au-BA nanoparticles on cell viability, T98G cells were cultured in 96-well plates at a density of 50.000 cells/well (100 μL per well), and they were grown in Dulbecco’s Modified Eagle’s medium (DMEM) supplemented with 10% fetal bovine serum (FBS), 100 U/mL penicillin, and 10 μg/mL streptomycin, in 5% CO_2_ in the air, at 37 °C. After 24 h of incubation, media were removed, and cells were treated with 0.1, 0.5, 5, and 10 μg/mL of MnFe_2_O_4_@PDA-Au-BA nanoparticles for 24 and 48 h. Untreated T98G cells were accepted as a control group. After the treatment of cells, media were removed, and an MTT (3-(4,5-dimethylthiazol-2-yl)-2,5-diphenyltetrazolium bromide) assay was performed. The formation of formazan crystals is used to determine viable cells using the colorimetric method.^54^ For the MTT assay, freshly prepared 100 μL of 0.5 mg/mL MTT was added to the well, and plates were incubated for 4 h at 37 °C in the dark. Then, the MTT reagent was removed and replaced with 100 μL of isopropanol to solubilize the converted purple dye in the wells. Absorbance at 570 nm was measured with an EnSight Multimode Plate Reader.

### 2.8 Statistical Analysis

Statistical analyses were performed using GraphPad Prism 5.01 for Windows (GraphPad Software). The viability of T98G cells in different MnFe_2_O_4_@PDA-Au-BA concentrations was compared using repeated measures variance analysis. The Friedman test was used to compare the viability in each concentration according to the control group, and *P* < 0.05 was taken at a significance level in all instances.

## 3 Results and Discussion

### 3.1 Preparation of Polydopamine-Coated MnFe_2_O_4_ Nanoparticles (MnFe_2_O_4_@PDA)

**Figure 2:**
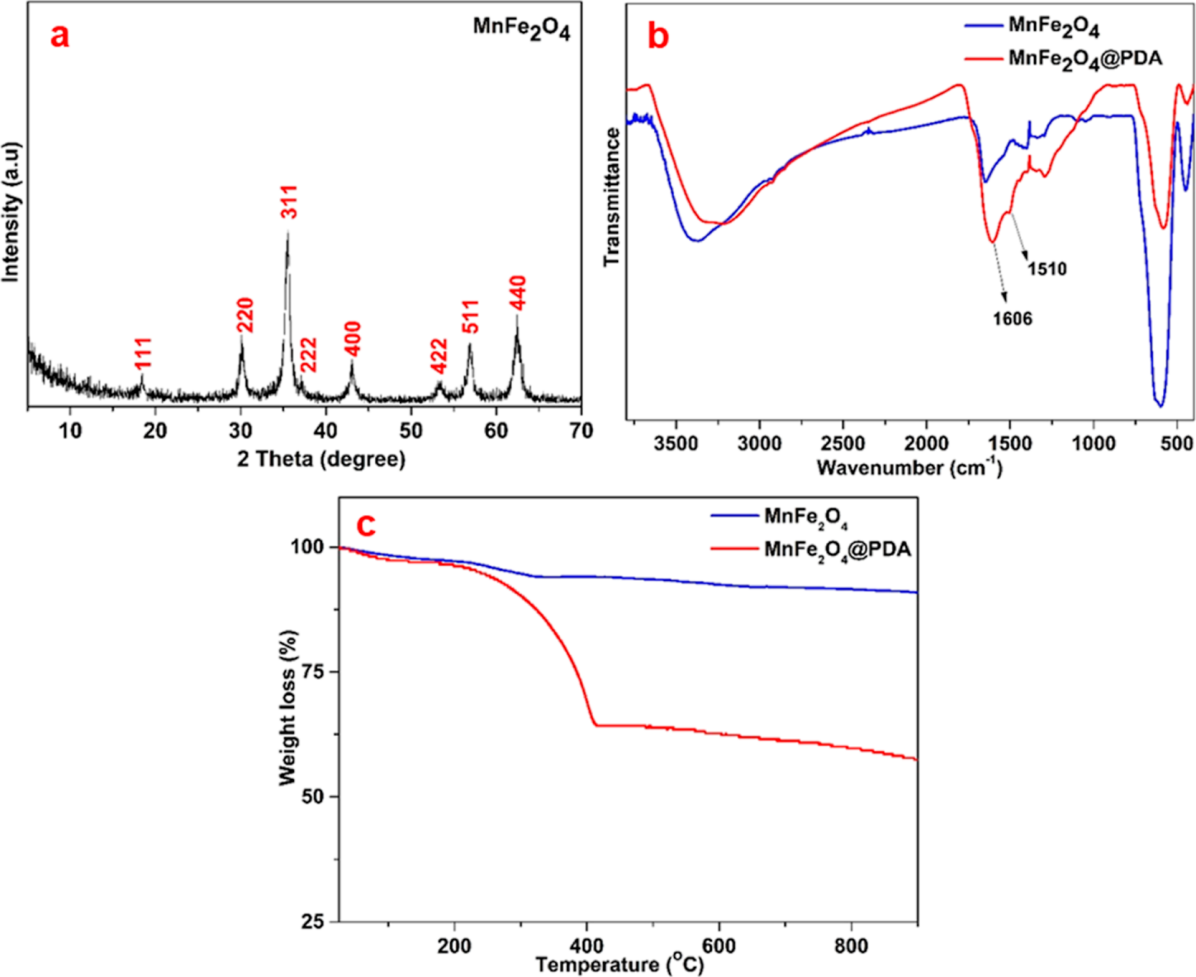
XRD pattern of MnFe_2_O_4_ nanoparticles (a), FT-IR spectra (b), and TGA curves (c) of MnFe_2_O_4_ and MnFe_2_O_4_@PDA nanoparticles.

The powder XRD pattern of MnFe_2_O_4_ nanoparticles prepared using the solvothermal method is shown in Figure 1a. The observed patterns at 18. 5° (111), 30.2° (220), 35.5° (311), 37.1° (222), 43.1° (400), 53.3° (422), 56.9° (511), and 62.5° (440) matched well with the standard card of MnFe_2_O_4_ (JCPDS card no. 74-2403), and no impurity phases or other product phases were observed in the pattern.^55,56^ After the synthesis of MnFe_2_O_4_ nanoparticles was confirmed by XRD analysis, these nanoparticles were coated with PDA, which not only can reduce metals (such as gold) in a solution to metal nanoparticles through catechol functional groups without the need for extra chemicals but also can increase the solubility of the whole structure in the biological environment by providing hydrophilic properties.^57^ In addition, boron atoms can be attached to the catechol groups of PDA by forming five-membered rings.^57^ Thus, PDA was chosen as the coating agent to modify MnFe_2_O_4_ nanoparticles for these reasons. Figure 2b indicates the FT-IR spectra of MnFe_2_O_4_ and MnFe_2_O_4_@PDA in the region of 450 to 4000 cm^–1^ to characterize functional groups. In the blue spectrum related to MnFe_2_O_4_ nanoparticles, the broadband at 3600–3000 cm^–1^ is attributed −to OH stretching vibration due to absorbed water molecules and the peaks at 1650 and 597 cm^–1^ belong to the C=O stretching of the residual PVP molecules and metal–O (Mn–O or Fe–O) bond vibrations of MnFe_2_O_4_ nanoparticles, respectively.^58^ The other spectrum of MnFe_2_O_4_@PDA nanoparticles indicates the new peaks at 1606 and 1510 cm^–1^, which belong to C=C stretching vibrations of a benzene ring and N–H bending vibrations, respectively, verifying the presence of PDA with MnFe_2_O_4_ nanoparticles.^20^ The confirmation and determination of the PDA coating were performed by TGA, as shown in Figure 1c. While MnFe_2_O_4_ nanoparticles showed a total weight loss of 10.3% due to absorbed water (2.3%) on the surface from 25 to 150 °C and residual PVP molecules (8%) from 150 to 1000 °C, MnFe_2_O_4_@PDA nanoparticles had 3 and 42.6% weight losses between the same temperatures. The difference in weight loss between 150 and 1000 °C was due to the removal of coated PDA from MnFe_2_O_4_ nanoparticles.

**Figure 3:**
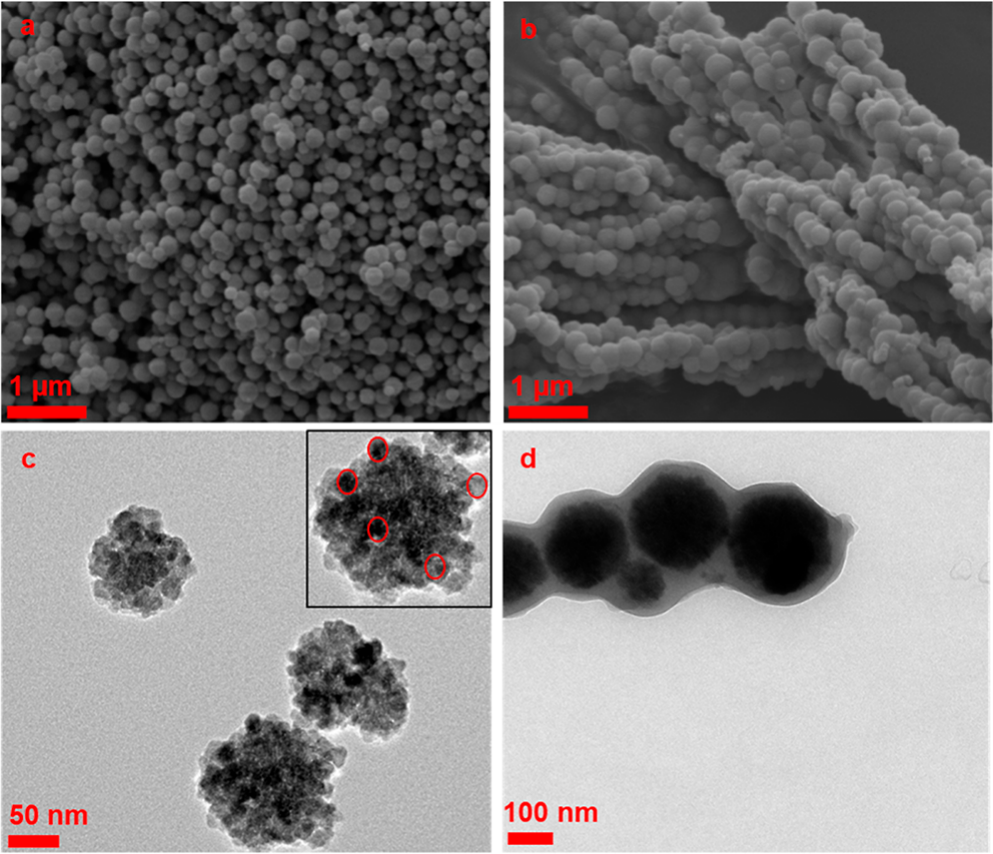
SEM and TEM images of MnFe_2_O_4_ (a,c) and MnFe_2_O_4_@PDA (b,d) nanoparticles.

SEM and TEM images performed to determine the morphology and size of the MnFe_2_O_4_ nanoparticles and confirm the PDA coating are shown in Figure 3. The SEM image in Figure 3a shows that MnFe_2_O_4_ nanoparticles consisted of a nearly spherical morphology with a size of 100–200 nm. In order to confirm that MnFe_2_O_4_ structures were composed of multicore nanoparticles, small-sized particles (approximately 100–130 nm) were selected from TEM analysis. MnFe_2_O_4_ structures appeared in bulk in the single nanoparticles with sizes ranging from 10 to 15 nm (Figure 3c). After the PDA coating, it was observed that the particle sizes ranged approximately between 180 and 280 nm (Figure 3b), and the PDA thickness was between 30 and 40 nm (Figure 3d).

### 3.2 Preparation of Gold-Loaded and Boron-Attached MnFe_2_O_4_@PDA Nanoparticles (MnFe_2_O_4_@PDA-Au-BA) and In Vitro Cell Viability of MnFe_2_O_4_@PDA-Au-BA

**Figure 4:**
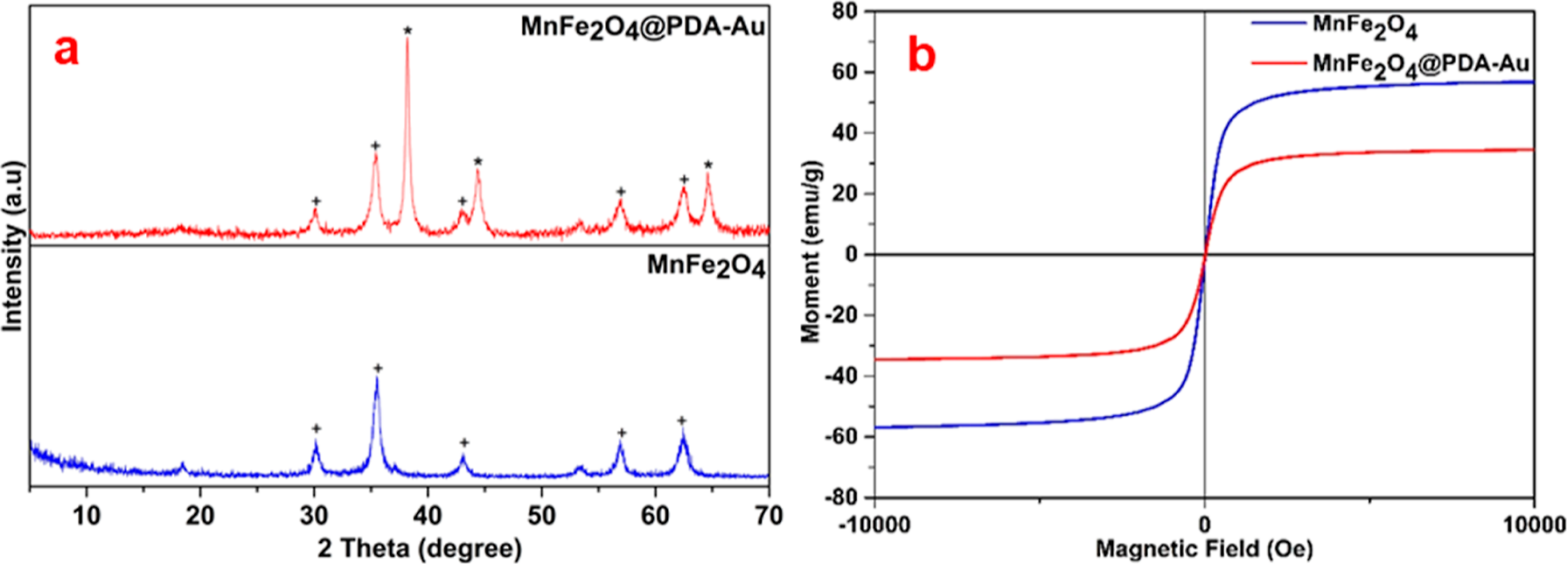
XRD pattern (a) and room-temperature magnetization curves (b) of MnFe_2_O_4_ and MnFe_2_O_4_@PDA-Au nanoparticles (+:MnFe_2_O_4_ and *:Au).

As detailed in the previous sections, the surface of MnFe_2_O_4_ nanoparticles was coated with PDA to reduce gold nanoparticles and attach boron atoms through catechol functional groups. The loading of gold nanoparticles to the MnFe_2_O_4_@PDA nanoparticles was first demonstrated by XRD analysis. In Figure 4a, the new phases seen at 38.1°(110), 44.5° (200), and 64.6°(220) exactly matched with the standard card of gold particles (JCPDS 04-0784). The phases related to MnFe_2_O_4_ did not change; only the intensity of the phases decreased due to the PDA coating. The room-temperature magnetization curves of MnFe_2_O_4_ and MnFe_2_O_4_@PDA-Au samples are shown in Figure 4b. The saturation magnetization (σs) values of MnFe_2_O_4_ and MnFe_2_O_4_@PDA-Au samples were calculated as 58.3 and 35.1 emu/g from the magnetization curves, respectively. As expected, the saturation magnetization value decreased after modifying non-magnetic PDA and gold nanoparticles, but the low coercivity, residual magnetism, and narrow hysteresis areas were related to the soft ferromagnetic properties. The soft ferromagnetic particles could be readily magnetized and demagnetized, allowing them to be used in various applications.^59^

**Figure 5:**
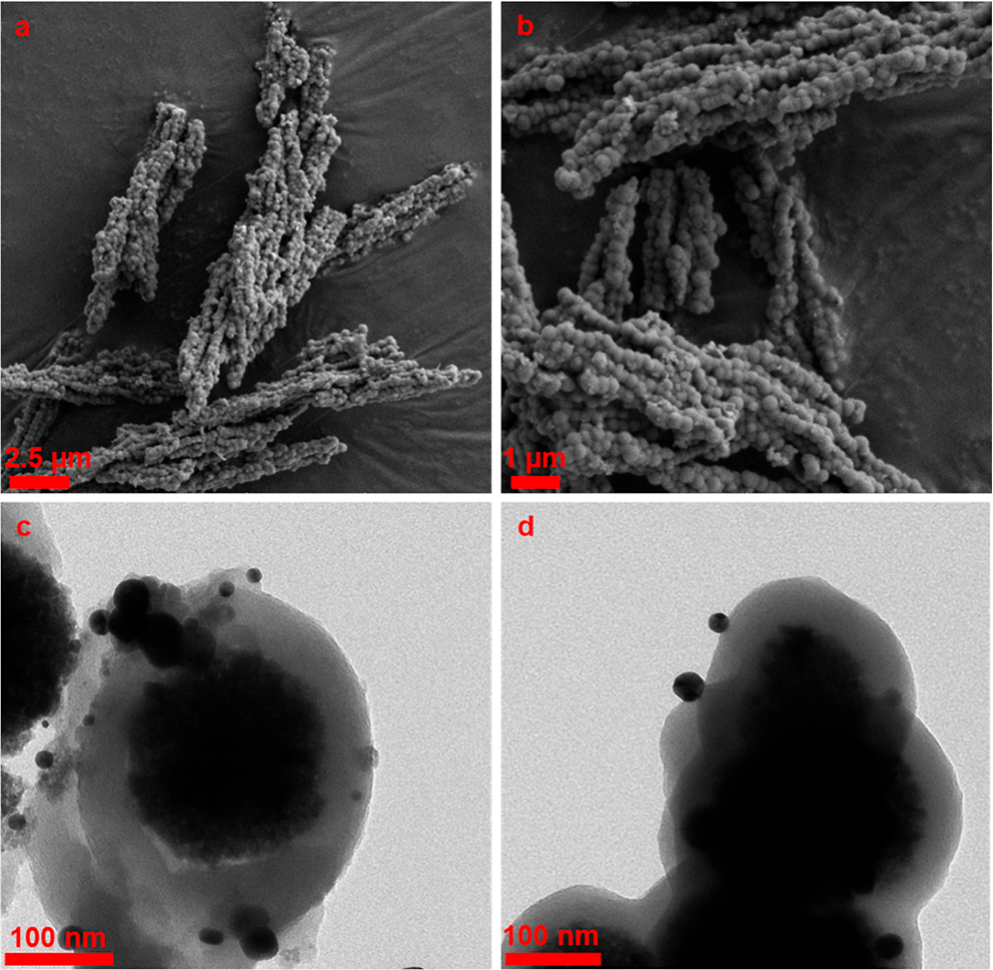
SEM (a,b) and TEM (c,d) images of MnFe_2_O_4_@PDA-Au nanoparticles.

Figure 5 shows the SEM (a,b) and TEM (c,d) images of MnFe_2_O_4_@PDA-Au nanoparticles. As can be seen from the SEM images, after loading the gold nanoparticles, the general morphology did not change and remained close to a spherical morphology, sized at nearly 180–280 nm, but small particles were formed on the surfaces. According to TEM analysis, it was confirmed that gold nanoparticles were formed on the PDA surface with a size of approximately 20–30 nm. Depending on the sample preparation for SEM and TEM analyses, MnFe_2_O_4_@PDA-Au nanoparticles appear to be aggregated in the solid form. However, different methods such as sonication and dispersing in various suspension environments can be applied for better dispersion of these nanoparticles in the biological environment.^60−62^

**Figure 6:**
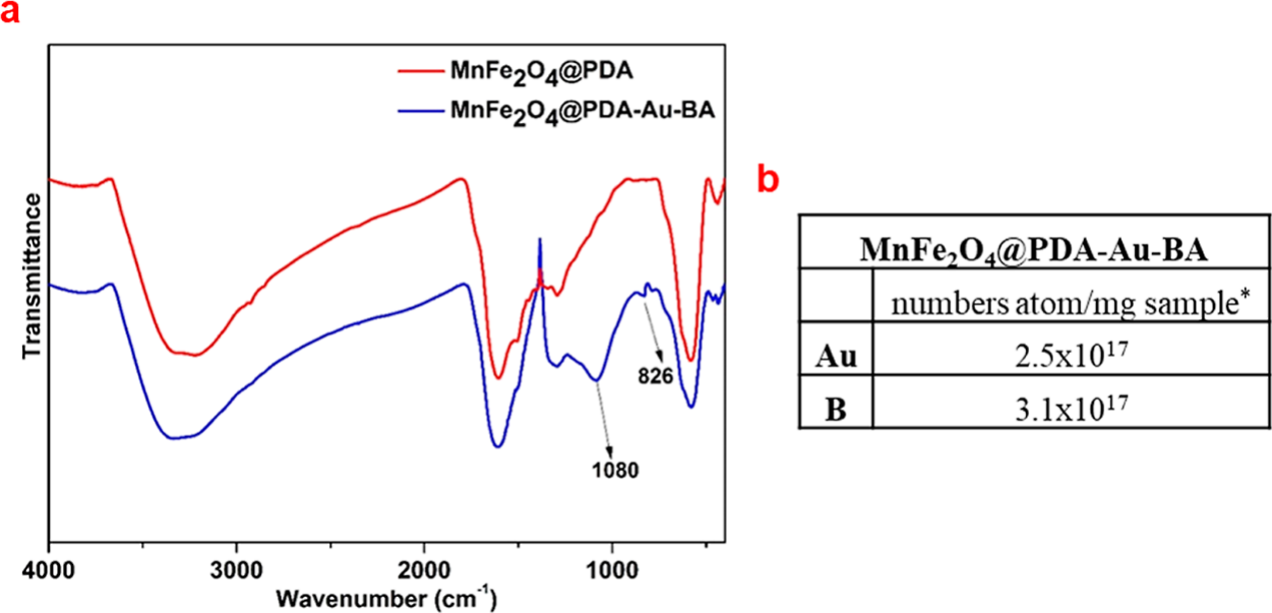
FT-IR spectra of MnFe_2_O_4_@PDA and MnFe_2_O_4_@PDA-Au-BA nanoparticles (a); Au, and B contents of MnFe_2_O_4_@PDA-Au-BA nanoparticles (b);* values found by ICP-OES analysis.

After the gold nanoparticles were reduced on the surface of MnFe_2_O_4_@PDA, boric acid was finally attached to MnFe_2_O_4_@PDA-Au via the catechol groups of PDA by forming five-membered rings. The final sample (MnFe_2_O_4_@PDA-Au-BA) was characterized by FT-IR and ICP-OES analyses shown in Figure 6. After boric acid attachment, the new peaks observed at 1080 and 826 cm^–1^ are related to asymmetric and symmetric B–O vibrations of the tetrahedral BO_4_ groups, respectively, indicating that boric acid is attached to PDA by converting the BO_4_ group.^47,63^ According to ICP-OES analysis (as shown in Figure 6b), the numbers of Au and B per milligram sample were calculated as 2.5 × 10^17^ and 3.1 × 10^17^ atoms/mg, respectively, which could make an appropriate candidate for magnetic targeted BNCT and PTT applications.^47,64^ When higher amounts are required for each application, the amounts of B and Au can be tuned by the PDA thickness.

**Figure 7:**
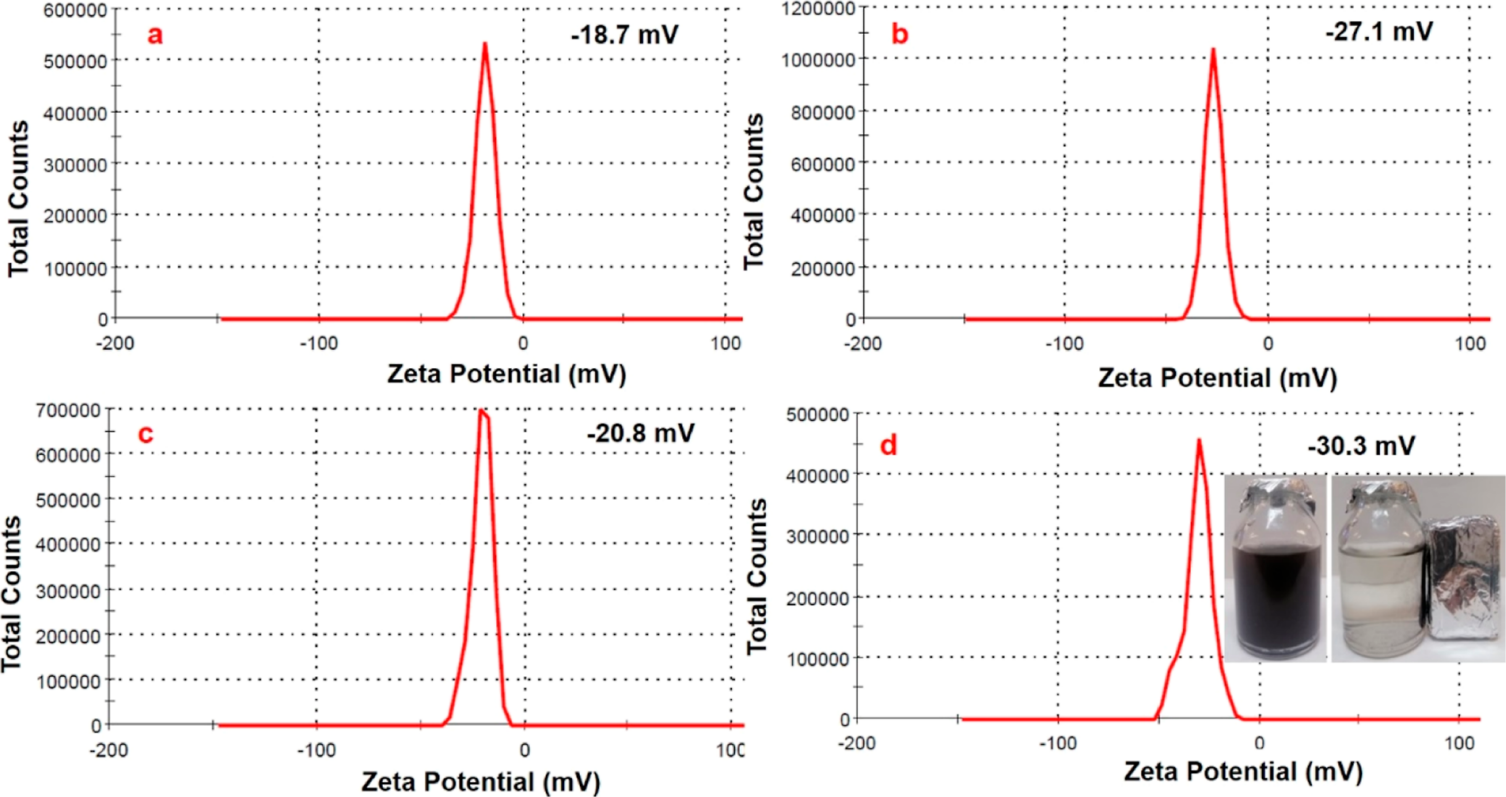
Zeta potential distributions of MnFe_2_O_4_ (a), MnFe_2_O_4_@PDA (b), MnFe_2_O_4_@PDA-Au (c), and MnFe_2_O_4_@PDA-Au-BA (d) dispersed in water.

Zeta potential measurements were also performed to confirm the surface modifications for all samples, as shown in Figure 7. Multicore MnFe_2_O_4_ nanoparticles had a negative zeta-potential of −18.7 mV depending on the PVP molecules, and after PDA modification (MnFe_2_O_4_@PDA), the negative zeta potential of the nanoparticles increased from −18.7 mV to −27.1 because of the deprotonation of the catechol group of the PDA. However, MnFe_2_O_4_@PDA-Au nanoparticles presented a zeta potential of −20.8 mV because the number of catechol groups of the PDA used during the reduction of gold nanoparticles to the surface was reduced. Finally, the negative zeta potential increased up to −30.3 mV owing to the negatively charged tetrahedral BO_4_ groups after boric acid attachment.^65−67^ Furthermore, it can be seen that MnFe_2_O_4_@PDA-Au-BA nanoparticles were dispersed uniformly in an aqueous colloidal system with black color, and the dispersed nanoparticles were easily attracted using external magnet (inset in Figure 7d).

**Figure 8:**
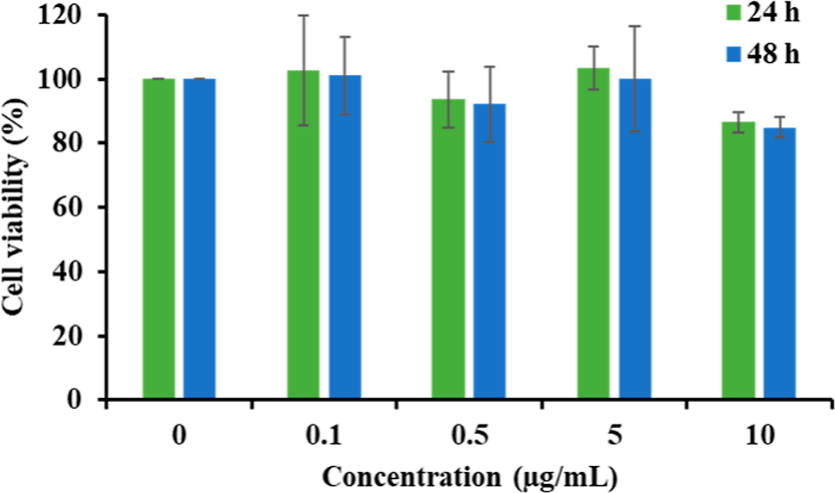
Graph of cell viability results of 24 and 48 h-treated T98G cells with MnFe_2_O_4_@PDA-Au-BA. Untreated T98G cells were set to 100% as a control. The graph was given as the mean ± S.D. of independent experiments (*n*: 3).

After the designed MnFe_2_O_4_@PDA-Au-BA was successfully characterized by various techniques, T98G cells were used to determine its effect on cell viability. After the treatment with different concentrations of MnFe_2_O_4_@PDA-Au-BA nanoparticles, data were analyzed, and variance analysis of repeated measures was performed using the Friedman test. According to the cell viability assay, treatment with MnFe_2_O_4_@PDA-Au-BA from 0.1 to 10 μg/mL during 24 and 48 h did not cause any significant cell death (Figure 8). The most obvious decrease in the percentages of cell viability was seen at the concentration of 10 μg/mL in both 24 and 48 h treatments (Figure 8), but they were not significant at all. The 48 h treatment caused slight decreases in cell viability than that of the 24 h-treatment for all concentrations; however, it was not significant in all instances. According to the cell viability assay, it can be said that MnFe_2_O_4_@PDA-Au-BA nanoparticles indicate good biocompatibility.

## 4 Conclusions

This study demonstrates that the surfaces of multicore manganese ferrite nanoparticles can be modified with gold and boron via the catechol groups of PDA. MnFe_2_O_4_ structures consisting of single nanoparticles with sizes ranging from 10 to 15 nm were prepared using the solvothermal method and verified with XRD analysis. Multicore MnFe_2_O_4_ structures were coated with PDA to provide the reduction of gold nanoparticles, attachment of boron on the surface, and the biocompatibility of all materials. MnFe_2_O_4_@PDA nanoparticles indicated the new peaks at 1606 and 1510 cm^–1^ in the FT-IR spectrum, which belong to C=C stretching vibrations of a benzene ring and N–H bending vibrations, respectively, and a total weight loss of 45.6% in the TGA curve confirmed the existence of PDA. In addition, the shell thickness was determined using TEM between approximately 30 and 40 nm. Au nanoparticles possessing a spherical morphology with sizes ranging from 20 to 30 nm were seen on the PDA surface after Au loading. The MnFe_2_O_4_@PDA-Au sample exhibited soft ferromagnetic properties (35.1 emu/g) with low coercivity, residual magnetism, and narrow hysteresis areas, which is suitable for biomedical applications. Finally, after boric acid attachment, the new peaks at 1080 and 826 cm^–1^ in the FT-IR spectrum related to asymmetric and symmetric B–O vibrations of the tetrahedral BO_4_ groups, respectively, confirmed that boric acid was attached to PDA by converting the BO_4_ group. According to the cell viability assay, treatment with MnFe_2_O_4_@PDA-Au-BA from 0.1 to 10 μg/mL during 24 and 48 h did not cause significant cell death. As a result, this designed multifunctional MnFe_2_O_4_@PDA-Au-BA may be a suitable candidate for various biomedical applications such as MRI, PTT, and BNCT.
